# Bleomycin Electrochemotherapy of Dermal Cylindroma as an Alternative Treatment in a Rare Adnexal Neoplasm: A Case Report and Literature Review

**DOI:** 10.3390/biomedicines11102667

**Published:** 2023-09-28

**Authors:** Antonio Bonadies, Alessandra Iorio, Vitaliano Silipo, Carlo Cota, Flavio Andrea Govoni, Michela Battista, Tiziano Pallara, Emilia Migliano

**Affiliations:** 1 Department of Plastic and Regenerative Surgery, San Gallicano Dermatological Institute—IRCCS, 00144 Rome, Italy; tiziano.pallara@ifo.it (T.P.); emilia.migliano@ifo.it (E.M.); 2 Department of Oncologic and Preventative Dermatology, San Gallicano Dermatological Institute—IRCCS, 00144 Rome, Italy; alessandra.iorio@ifo.it (A.I.); vitaliano.silipo@ifo.it (V.S.); 3 Dermatopathological Laboratory, San Gallicano Dermatological Institute—IRCCS, 00144 Rome, Italy; carlo.cota@ifo.it; 4 Department of Maxillofacial Surgery, ACO San Camillo, Forlanini, 00152 Rome, Italy; fgovoni@scamilloforlanini.rm.it; 5 Scientific & Medical Department, IGEA S.p.A, 41012 Carpi, Italy; m.battista@igeamedical.com

**Keywords:** rare skin tumor, dermal cylindroma, electrochemotherapy, bleomycin, Brooke–Spiegler syndrome, adnexal neoplasm

## Abstract

Background: Brooke–Spiegler syndrome is a rare autosomal dominant disorder characterized by the continuous development of multiple benign skin appendage tumors. It is treated usually by repeated standard surgery. Here, we present a case study where electrochemotherapy (ECT) with bleomycin was used as an effective alternative approach in treating advanced dermal cylindromatosis of the head and neck in a patient with Brooke–Spiegler syndrome. Patients and methods: A 45-year-old woman presented with multiple recurrent dermal cylindroma lesions on her scalp. Previous treatment consisted of several surgical excisions that resulted in psychological deterioration due to the formation of numerous scars and extensive alopecic areas. ECT was offered to provide tumor removal and disease control and to improve the patient’s quality of life. Results: The treatment was well tolerated, and a significant reduction in neoplastic tissue was achieved. Importantly, scalp skin condition significantly improved, regaining a fair follicular density on the margins. Conclusion: This report suggests the feasibility of bleomycin ECT as a less invasive alternative option for controlling multiple scalp cylindroma lesions with cosmetically acceptable results, and improving quality of life.

## 1. Introduction

Brooke–Spiegler syndrome (BSS) is a rare autosomal dominant disorder due to various mutations in the *CYLD* gene on chromosome 16q12-q13 [1]. Typically, this syndrome manifests after puberty, in adolescence and early adulthood, with the development of multiple benign skin appendage tumors, including cylindroma, spiradenoma, and trichoepithelioma. After the appearance of the first lesion, tumors progressively increase in size and number and accumulate throughout life. BSS seems to have greater expressivity in women, generally affecting several family members of the same generation [2]. Cylindromas are pink, smooth-surfaced, nodular tumoral lesions varying in size from a few millimeters to several centimeters; they can present as single or clustered nodules. Occasionally painful, these lesions mostly affect the scalp, face, and neck, but can also be found on the trunk and upper limbs. Multiple scalp lesions may confluence to form a ‘turban tumor’ lesion [3]. The malignant transformation of cylindromas is rare, but metastases in cases of malignancy are not uncommon [4]. Cylindromas are usually removed by radical surgical excision. Repeated surgical procedures are often necessary to control tumor burden, especially due to the high rate of recurrence. Furthermore, in a functional and aesthetic setting, the scalp and skin should be preserved as much as possible [5].

Electrochemotherapy (ECT) is a non-thermal local ablative treatment for solid neoplasms [6]. It is based on the combination of reversible electroporation (EP), the application of short, intense electric pulses, and the administration of a chemotherapeutic agent. Due to increased cell membrane permeability, the otherwise non-permeable chemotherapeutic agent can enter the cell; combining EP with chemotherapy generates a superior synergistic local cytotoxic effect. The most suitable drug for the clinical use of ECT is bleomycin, an antineoplastic agent frequently used to treat head and neck cancer [7]. The mechanism of action of bleomycin is to generate single- and double-stranded DNA breaks that lead to DNA fragmentation, chromosomal gaps, and deletions, and ultimately to mitotic cell death [8]. ECT exploits the presence of bleomycin in the target tumor at the time of electric pulse application to enhance the drug’s efficacy locally [9]. ECT has been shown to be highly effective in providing local tumor control and palliation of symptomatic lesions located in cutaneous or subcutaneous tissues, regardless of their histological origin, while maintaining a very low toxicity profile and high patient acceptance [10,11]. ECT is used as a neoadjuvant therapy with cytoreductive function: as a therapeutic option in unresponsive patients and/or when conventional treatments are not performed, and as a palliative therapy for highly vascularized nodules [12,13]. Further clinical evidence supports early ECT application to treat smaller lesions, improve efficacy, and eradicate multiple target nodules in a single treatment session, thus reducing the therapeutic burden on the patient [11].

Here, we present a case of a patient with multiple cylindromas of the scalp treated with bleomycin ECT, resulting in effective control of the disease with good aesthetic results. Therefore, we propose bleomycin ECT as an alternative therapeutic approach in BSS for benign skin appendage tumors, such as cylindromas.

## 2. Case Report

In December 2017, a 45-year-old woman presented at the Department of Plastic Surgery of San Gallicano Institute for the evaluation of multiple cancerous lesions on her scalp. The patient reported that the first lesion arose on her scalp at the age of 18 years, and, over time, several more lesions appeared and progressively increased in number and dimension, developing extensively on her face, trunk, and extremities. Since then, the patient underwent multiple surgical excisions at several locations, including the dorsal region of the trunk, the face in the preauricular region, and the scalp, in order to remove the nodules and minimize the aesthetic problem and physical discomfort caused by them. While effective in eliminating the nodules, the surgical treatments were all less aesthetically successful, leading to psychological deterioration due to the inevitable formation of numerous scars or multiple hypo/hyperpigmented scarring outcomes.

The first clinical examination of the patient at our institution showed about 60 rosy red, single, clustered, or confluent, painless nodular scalp lesions of variable size, ranging from 1 to 3 cm in diameter, with a firm-elastic consistency, mobile on the superficial and deep planes. On the face (glabellar, peri-nasal, and preauricular region) and the trunk (back and décolleté), there were numerous asymptomatic, small (2–5 mm in diameter) skin-colored papules. No malignant transformation features were noted. Based on clinical and histopathological evidence, the patient was diagnosed with BSS in 1990, when she was eighteen years old, and she removed the first lesion.

After receiving informed consent from the patient, tissue biopsies were obtained from four nodular lesions of the scalp and one small papule in the perinasal region. The histopathological analysis of the four nodules revealed typical features of a cutaneous cylindroma, and the examination of the small papule showed histological features of trichoepithelioma, overall confirming the diagnosis of BSS.

**Figure 1:**
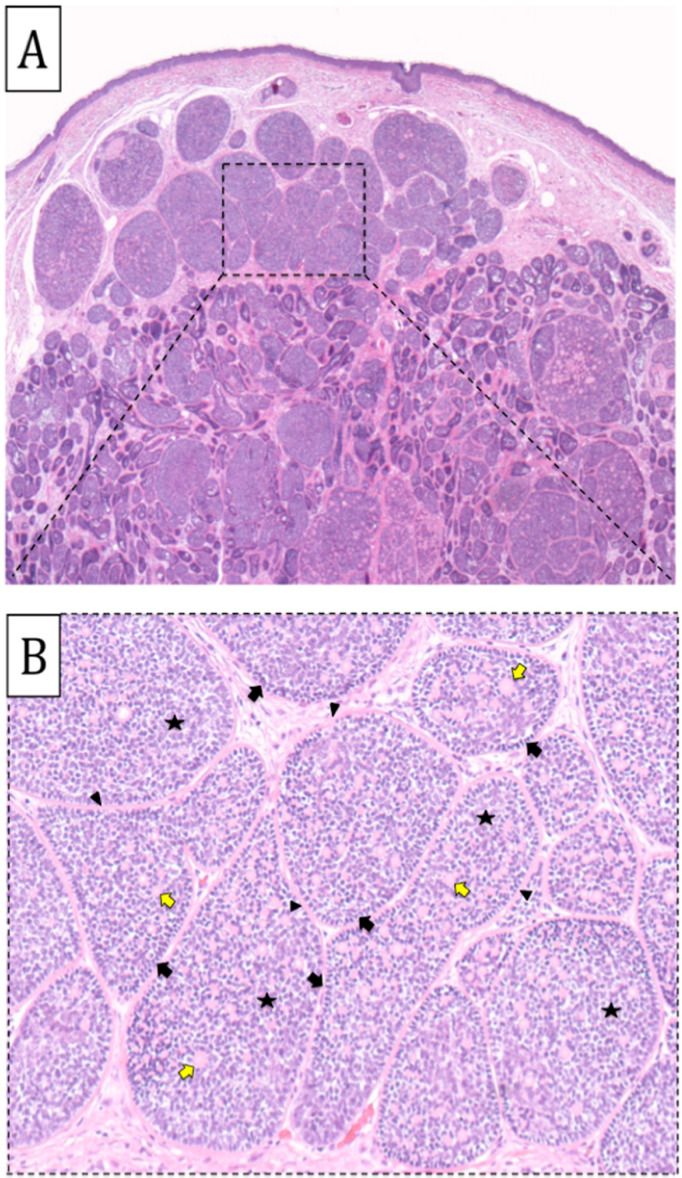
**Histopathological analysis of the scalp lesion before ECT treatment (H&E stain).** The dermal proliferation of basaloid cells arranged in a nest with the typical “jigsaw” puzzle pattern compatible with cylindroma (×40) (**A**). Higher magnification of the dotted rectangle region (×200) shows that each nest is composed of a palisading peripheral lining of smaller basophilic cells (black arrows), an inner population with larger and more differentiated paler cells (stars), and shows the presence of eosinophilic hyaline-like globules (yellow arrows). The nests are surrounded by a thickened eosinophilic hyalinized basement membrane material (arrowheads) (**B**).

At that time, the extent of disease at the scalp site would have required the excision of large areas of the scalp, followed by reconstruction with dermo-epidermal grafts, large locoregional flaps, or a free flap. This is the reason for the idea of using ECT with bleomycin to test the response of cylindromas and evaluate the disease control rate, considering that the progressive growth of such lesions can turn into malignant transformation. ECT with bleomycin was offered as a less invasive alternative option to ensure tumor removal while avoiding further scarring and unpleasant cosmetic results. Another goal was to improve the patient’s quality of life (QoL) by reducing the number of surgical procedures and hospital visits. In March 2018, three large areas of the scalp with a high number of clustered or confluent lesions and a pre-auricular lesion on the face were treated with the first bleomycin ECT session at the operating block of San Gallicano Institute, IRCCS. The procedure lasted twenty minutes under mild sedation according to the European Standard Operating Procedures on Electrochemotherapy [14]. Briefly, bleomycin (Sanofi-Aventis S.p.A., Milano, Italy, vials of 15 mg) was administered intravenously at a dose of 15,000 UI/m^2^, followed by local application of high-voltage electrical pulses to each single tumor nodule. Type III hexagonal configuration electrodes were used. The electrical pulses (eight pulses of 100 ms duration) were delivered using a square-wave pulse generator (Cliniporator, IGEA S.p.A., Carpi, Italy). During the ECT procedure, a biopsy of one lesion (8 × 8 mm) was taken before treatment. A histopathological analysis showed that the multinodular lesion located in the scalp dermis was composed of small basaloid cells intermixed with paler cells with few lymphocytes. The presence of eosinophilic hyaline-like globules was evident. The epithelial cells were arranged as typical trabeculae/nests in a jigsaw puzzle pattern compatible with the cylindroma (Figure 1A). At greater magnification, the classic monomorphism of basophilic cells bordered by a thickened hyaline basal membrane was appreciated; the hyaline material, which exhibited a labyrinthine aspect in some areas, was interposed between the lobular structures (Figure 1B). All treated lesions were recorded with photographs to evaluate the aesthetic and functional results after treatment. The procedure was well tolerated, and no serious bleomycin ECT-related adverse events, such as pulmonary toxicity, were observed. Moderate facial edema was recorded in the postoperative period and treated with oral corticosteroids, showing resolution within 72 h. Other side effects included the following: moderate pain, well controlled with paracetamol 1000 mg, max three tablets per day; minimal skin toxicity with rare superficial ulcerations and modest alopecia at the margins of the treated areas. Daily dressings were performed by cleansing with a solution based on water-soluble sulfur, acetic acid, and *Melaleuca alternifolia*, and then treated with a spray based on hyaluronic acid sodium salt 0.2% (aluminum starch octenylsuccinate, hexamethyldisiloxane, sweet almond oil, vitamin E acetate, ximenynic acid, EO lemon, EO *Melaleuca alternifolia*, hyaluronic acid sodium salt, butane, isobutane, propane).

At 2 months following the first treatment, an objective response was observed in all the treated areas, with complete response achieved in 25% of the treated lesions and partial tumor regression (≥30% reduction in tumor size according to the RECIST criteria) in the remaining lesions. In November 2018, eight months after the first treatment, the patient underwent a second ECT session on the previously treated partially responsive nodules, and new lesions developed outside the treated area. The second treatment involved all lesions present on the scalp, face, and trunk.

Moderate edema, pain, skin ulceration, and alopecia were also detected after the second treatment session. Additional hyperpigmentation was observed mainly on the face and trunk and was managed with the application of lactoferrin-based iron-chelating creams and/or EDTA + vitamin C, with marked improvement after several months of application.

**Figure 2:**
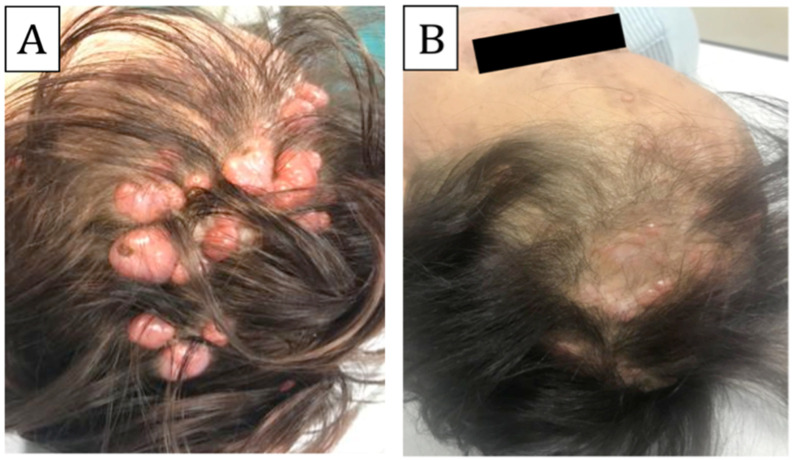
**Multiple cylindroma lesions localized on the scalp treated with bleomycin ECT.** Baseline presentation before starting bleomycin ECT (**A**); at 1 year after bleomycin ECT (**B**).

In April 2019, at 13 months after the first ECT treatment (5 months after the second session), a further reduction in neoplastic tissue was achieved by the sum of the effects of both treatments (Figure 2). Unfortunately, new lesions also appeared during this time because of the natural history of the disease, but no areas of malignant transformation were detected. Moderate scarring alopecia was observed in limited areas. If, before the treatment, the scalp appeared erythematous, reddish-pink, tight, thin, and alopecic, it regained a nearly normal skin tone, thickness, and elasticity in the post-treatment evaluation. Although it remained alopecic in the central areas, it recovered a fair follicular density on the margins.

This case report was approved by the central Ethical Committee IRCSS Lazio under the n° 1449/20. Written informed consent was obtained from the patient for the publication of this case report and any accompanying images.

## 3. Discussion

Cylindromas are very rare adnexal tumors, most frequently localized on the head and neck and often linked to Brooke–Spiegler syndrome. This report documents the safety and efficacy of bleomycin ECT in a patient with multiple benign skin adnexal tumors of the Brooke–Spiegler type. The treatment methods reported in the literature for cylindromas are mostly repeated non-demolishing surgical procedures. However, multiple scars from the repeated scalp excisions necessary to remove multiple lesions or large scars from extensive scalp resections can profoundly impact patients’ QoL [5]. Complete scalp resection followed by skin graft reconstruction is used for patients with familial cylindromatosis who are unresponsive, or unsuitable for other treatments. Although this strategy abolishes any possibility of the recurrence of scalp lesions, this treatment leads to complete scalp alopecia [15,16]. “Scalp-sparing” strategies include early primary excision and tumor enucleation [17], with a risk of recurrence after first-line treatment of about 35%. Mohs micrographic surgery is recommended for sporadic recurrent cases [5]. Hyfrecation or repeated laser treatment can be applied in selected small tumors [15,18]. Radiation therapy has also been proposed to treat this disorder, but patients with BSS may not benefit from its use. Several studies in the current literature have shown that this method may increase the risk of radiation-induced DNA damage and malignant transformation, and is therefore contraindicated [1,3,4]. Other reported therapeutic options include dermabrasion, cryotherapy, and radiofrequency [15], electrocoagulation, intralesional infiltration with triamcinolone acetonide, topical phenol, both topically and systemically applied acetylsalicylic acid, and topical 5% imiquimod cream [5,16]. However, most of these therapies can lead to scar formation and often have high recurrence rates. To date, most treatment strategies for scalp cylindromas have the disadvantage of leading to scar formation and functional impairment. Permanent alopecic areas where scar tissue replaces hair follicles can lead to significant changes in physical appearance, and, thus, psychological distress, especially in patients with a thick head of hair, as seen in the patient in this case report.

For these reasons, finding new or existing treatment modalities that have a selective, non-invasive effect on tumor tissue is essential. The efficacy of ECT has been largely reported in a wide variety of cutaneous and subcutaneous tumors, including non-melanoma skin cancers of the head and neck, such as squamous cell, basal cell, and Merkel cell carcinoma, melanoma metastases, and Kaposi sarcoma [10,11,19]. Because the cytotoxic effect of ECT is mostly limited to dividing tumor cells, in some cases, ECT may be both an organ- and a function-sparing modality [20] that allows for favorable aesthetic results [21]. Furthermore, ECT is a repeatable treatment with an associated increase in response rate [22]. To our knowledge, this is the first case report in which bleomycin ECT is proposed as an efficient and scalp-sparing alternative strategy in managing multiple cylindromas of the scalp. At the time of admission to the clinic, the patient would have needed an excision of extensive areas of the scalp. Instead, the choice of bleomycin ECT treatment was shown to be able to provide disease control. The treatment resulted in a cosmetically acceptable outcome, healthier skin appearance, limited scarring alopecic areas, and good preservation of hair follicles. In our case, using bleomycin ECT also improved the patient’s QoL by greatly reducing the hospitalization time and the number of repeated removal procedures. Longer follow-up and more extensive experience are needed to corroborate the value of this new approach. Although the rarity of the disease precludes broader observations, this case report presents a valuable strategy that could help to manage patients with BSS.

## 4. Conclusions

Bleomycin ECT represents an effective scalp-sparing alternative treatment for scalp cylindroma in patients with Brooke–Spiegler syndrome. A significant improvement in QoL was achieved, and the strategy reduced scarring and preserved hair follicles. Considering the results achieved in this case, the authors suggest that this method can be applied also in cases of large lesions of other anatomical areas, where surgical excision may damage function and/or aesthetics.

## Data Availability

All data generated and analyzed during this study are available within the paper. Further inquiries can be directed to the corresponding author.
